# Different Airway Inflammatory Phenotypes Correlate with Specific Fungal and Bacterial Microbiota in Asthma and Chronic Obstructive Pulmonary Disease

**DOI:** 10.1155/2022/2177884

**Published:** 2022-03-11

**Authors:** Rui Yang, Qiao Zhang, Zhidong Ren, Hong Li, Qianli Ma

**Affiliations:** ^1^Institute of Respiratory Diseases, Second Affiliated Hospital, Third Military Medical University, Chongqing, China; ^2^Center for Chronic Respiratory Disease Management, North-Kuanren General Hospital, Chongqing, China

## Abstract

**Background:**

Studies of chronic airway inflammatory diseases have increasingly focused on airway microbiota. However, the microbiota characteristics of asthma and chronic obstructive pulmonary disease (COPD) patients with different airway inflammatory phenotypes remain unclear.

**Objective:**

We aimed to reveal the differences of fungal and bacterial microbiota between eosinophilic asthma (EA) and noneosinophilic asthma (NEA) patients and between eosinophilic COPD (EC) and noneosinophilic COPD (NEC) patients. Further, explore whether similarities exist in the airway microbiota of patients with the same phenotype.

**Methods:**

Induced sputum samples were collected from 45 asthma subjects and 39 COPD subjects. The airway microbiota of the subjects was profiled by nearly full-length 16S rRNA and internal transcribed space (ITS) sequencing.

**Results:**

Subjects with eosinophilic phenotype (EA and EC) showed significant differences in both fungal and bacterial microbiota compared to the corresponding subjects with noneosinophilic phenotype (NEA and NEC). In addition, no differences were observed between the fungal microbiota of subjects with the same phenotype (EA vs. EC, NEA vs. NEC). In bacterial microbiota, the greater relative abundance of *Streptococcus thermophilus* was observed in EA and EC subjects, while Ochrobactrum was enriched in NEA and NEC subjects. In fungal microbiota, the EA and EC subjects showed higher relative abundances of *Aspergillus* and *Bjerkandera*, while the NEA and NEC subjects were enriched in *Rhodotorula* and *Papiliotrema*.

**Conclusions:**

Different airway inflammatory phenotypes were related to specific fungal and bacterial microbiota in both asthma and COPD, while the same airway inflammatory phenotype revealed a degree of similarity in airway microbiota, particularly in fungal microbiota.

## 1. Introduction

Over 500 million people worldwide are affected by the prevalence of chronic airway inflammatory diseases and consume substantial public health resources, represented by asthma and chronic obstructive pulmonary disease (COPD) [1]. Both of them are heterogeneous chronic airway inflammatory diseases with airflow limitations, and the common pathological bases are airway remodeling and airway inflammation dominated by inflammatory cell infiltration. It is widely recognized that airway inflammatory phenotypes in both asthma and COPD were divided into eosinophilic phenotype and noneosinophilic inflammatory phenotypes according to the level of peripheral blood and/or sputum eosinophil count. T helper (Th) 2 lymphocyte-mediated eosinophilic inflammation is the representative airway inflammation of approximately 50% of asthma patients [2]; also, increased airway eosinophilic inflammation can be observed in around 10%-40% of COPD patients [3, 4].

Recently, the associations between airway inflammatory phenotypes and microbiota are receiving increasing attention with the development of culture-independent technology. Studies had shown the distinct structure of bacterial microbiota in patients with higher eosinophilic inflammation, compared with patients with lower eosinophilic inflammation and healthy controls, in both asthma and COPD [5–9]. Furthermore, the fungal microbiota also plays a key role in airway inflammatory diseases. Sharma et al. [10] demonstrated that asthma patients with high/low blood eosinophils differ in fungal microbiota composition and the specific genera were associated with clinical parameters. However, the relationship between eosinophilic inflammation and fungal microbiota in subjects with COPD was poorly understood. Further, it is not clarified about the similarities and differences in the effects of eosinophilic phenotype on the microbiota of asthma and COPD.

In this study, we sought to explore the differences in the fungal and bacterial microbiota associated with eosinophilic and noneosinophilic inflammatory phenotypes in stable COPD and asthma patients. Through the nearly full-length 16S rRNA and internal transcribed space (ITS) sequencing, we reported that the fungal microbiota of eosinophilic and noneosinophilic inflammatory phenotypes was distinct in subjects with COPD and further found the fungal microbiota was similar in asthma and COPD subjects with the same airway inflammatory phenotype.

## 2. Study Design and Subjects

Subjects in this study were from the first visit of a prospective observational cohort study, which was approved by the Medical Ethics Committee of the Second Affiliated Hospital of the Army Medical University and registered in Chinese Clinical Trial Registry (http://www.chictr.org.cn, No.: ChiCTR1900024871). In accordance with the Declaration of Helsinki, the written informed consent was provided by all subjects before enrollment in the study. The induced sputum samples were collected from adult subjects with stable COPD or asthma.

Subjects with COPD were diagnosed based on the 2019 Global Initiative for Chronic Obstructive Lung Disease (GOLD) guidelines [11], the postbronchodilator forced expiratory volume in 1 s/forced vital capacity ratio (FEV_1_/FVC) < 0.7 and FEV_1_ < 80% predicted. Asthmatic subjects were diagnosed according to the 2019 Global Initiative for Asthma (GINA) guidelines [12], with FEV_1_ increase > 12% and 200 ml after inhaling 400 *μ*g salbutamol or a positive bronchial provocation test result. Exclusion criteria of subjects included acute exacerbations in the last 3 months; antibiotic or antifungal drug usage within 4 weeks before recruitment; subjects with any other chronic respiratory disease that may interfere with optimal conditions for the study, including (but not limited to) patients with asthma-COPD overlap, active tuberculosis, bronchiectasis, interstitial lung disease, and cystic fibrosis.

Demographic and clinical characteristics of subjects were collected during the patient visits. COPD Assessment Test (CAT) and Asthma Control Test (ACT) were employed to evaluate the symptoms of subjects. Subjects were defined as eosinophilic asthma (EA)/COPD (EC) group if their sputum eosinophil percentage was ≥3%; otherwise, they were classified into noneosinophilic asthma (NEA)/COPD (NEC) group.

## 3. Samples' Collection and DNA Extraction

The samples' collection and processing process were based on our previous publications [13]. The plugs were attentively dispersed in dithiothreitol at 37°C. Trypan blue and hemocytometer were used to measure total cell counts. Cell count of leukocytes was counting at least 400 cells based on a stained with Wright-Giemsa slide. The sputum squamous cell percentages > 10% subjects were removed to reduce oral contamination. DNA extraction was performed with TIANamp DNA Kit (DP302-02, TIANGEN, Beijing, China). After determining the concentration and purity of products using NanoDrop ND2000 (Thermo, USA), the pure products were stored at -20°C before sequencing.

## 4. The Nearly Full-Length Sequencing and Bioinformatics Analysis

For bacteria, PCR amplification of the 16S rRNA genes was performed using the forward primer 27F (5′-AGAGTTTGATCMTGGCTCAG-3′) and the reverse primer 1492R (5′-ACCTTGTTACGACTT-3′) [5, 14]. Fungal ITS genes were performed using primers ITS1F (5′-CTTGGTCATTTAGAGGAAGTAA-3′) and LR3 (5′-CCGTGTTTCAAGACGGG-3′) [15–17]. The nearly full-length sequencing of 16S rRNA and ITS genes was carried out according to PacBio Sequel Systems powered by Single Molecule, Real-Time (SMRT) sequencing technology. PacBio circular consensus sequencing (CCS) reads were obtained by the multiple alignments of subreads to reduce sequencing error rates. Raw sequences were processed by SMRT Link portal; the CCS reads were considered as noise and were filtered out with minimum of 3 passes and a minimum predicted accuracy of 99%. Both fungi and bacteria sequencing data were processed using Quantitative Insights Into Microbial Ecology 2 (QIIME2) pipeline [18]. After chimera detection, the VSEARCH was employed to cluster high-quality sequences into operational taxonomic units (OTUs) at 97% similarity. A representative sequence was generated from each OTU using the default parameters, and the singleton OTUs were removed. To obtain a stricter taxonomic classification of each OTU, the BROCC algorithm [19] was performed after searching the nt database from NCBI using BLASTn, with the algorithmic threshold set to 100%. Procedural controls for the DNA extraction and PCR steps were included in the sequencing, and none of the four reagent control samples provided more than 100 read counts.

We measured the *α*-diversity (diversity within a habitat) of the microbiota using the Chao1 and Simpson indexes, the former to describe species richness, which refers to the number of species in a community or habitat, and the latter to describe species diversity, which is a combined measure of species richness and species evenness. The Wilcoxon rank-sum test and Dunn test were used to compare *α*-diversity between groups. Nonmetric multidimensional scaling (NMDS) [20] plots based on Bray-Curtis distance and permutational multivariate analysis of variance (PERMANOVA) [21] were employed to reveal *β*-diversity (diversity among habitats) variations. The differential microbiota features were identified using linear discriminant analysis effect size (LEfSe) [22] with LDA threshold set to 3.0, and taxa with relative abundances less than 0.5% were filtered out. Random forest models [23] were established against 10-fold cross-validation to supplement the biomarkers of fungal microbiota. Fungal-bacterial correlation networks were constructed based on Spearman's correlation coefficient (FDR-corrected *P* < 0.01 and ∣*r* | >0.5) between the top 20 genera. The networks were divided into modules using the igraph package [24] in R (3.6.1) software and visualized by Cytoscape (3.7.2) software. After the Kolmogorov-Smirnov test for normality of clinical characteristics, Fisher's exact test was used to test count data and Wilcoxon rank-sum test or *t*-test for measurement data.

## 5. Result

### 5.1. Enrolled Subjects' Characteristics

**Figure 1:**
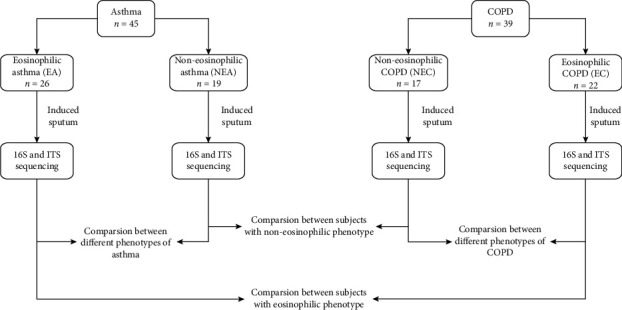
The study flowchart.

We collected 84 induced sputum samples from 45 asthma subjects and 39 COPD subjects. According to the percentage of sputum eosinophils, the asthma subjects were divided into the EA group (*n* = 26) and the NEA group (*n* = 19), and the subjects with COPD were divided into the EC group (*n* = 22) and the NEC group (*n* = 17) (Figure 1).

The NEA subjects had higher inhaled corticosteroid (ICS) doses than EA subjects, but this difference was not observed between EC and NEC subjects (Table 1). Furthermore, in comparisons between two groups with the same inflammatory phenotype (EA vs. EC, NEA vs. NEC), subjects with COPD had a higher proportion of males, were older, used higher doses of ICS, and had poorer lung function compared to the corresponding subjects with asthma.

### 5.2. Fungal Microbiota

**Figure 2:**
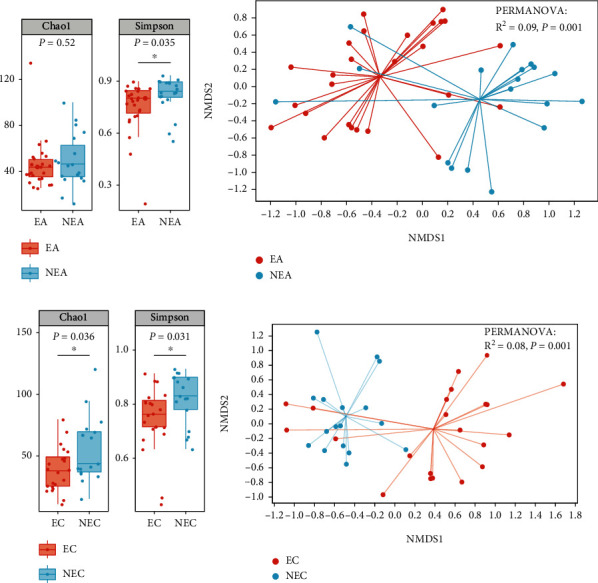
Alpha diversity and beta diversity of fungal microbiota. Each dot reprented a sample. Box plots of the Chao1 index and Simpson index, to compare alpha diversity (a) between EA and NEA and (c) between EC and NEC. The *y*-axis represented the value of the corresponding Chao1 or Simpson index. The statistical analysis was performed using Wilcoxon rank-sum test and Dunn' post hoc test. NMDS analysis based on the Bray-Curtis distance to compare beta diversity (b) between EA and NEA and (d) between EC and NEC. PERMANOVA was employed to reveal beta diversity variations.

The overviews of sequencing results are shown on supplementary material (Tables S1–S3). The Simpson (*P* = 0.035) index of the EA subjects was significantly lower compared that of NEA subjects (Figure 2(a)), which was consistent with previous studies in the asthmatic subjects [10, 25]. A comparable result had been observed in COPD subjects; the EC subjects showed lower Chao1 index (*P* = 0.036) and Simpson index (*P* = 0.031) than NEC subjects (Figure 2(c)). However, no significant difference in *α*-diversity was observed in asthma and COPD subjects with the same inflammatory phenotype (Figures S1A and S1C).

NMDS analysis based on the Bray-Curtis distance showed significant differences in *β*-diversity between the eosinophilic and noneosinophilic inflammatory phenotypes. In asthma subjects, the *β*-diversity was significantly different between the EA and NEA groups (Figure 2(b), PERMANOVA, *R*^2^ = 0.09, *P* = 0.001), and similar result was also observed in COPD subjects (Figure 2(d), PERMANOVA, *R*^2^ = 0.08, *P* = 0.001). The NMDS plots containing all samples and the Venn plots demonstrating OTUs shared between groups were displayed in the supplemental file (Figure S1B). Interestingly, there was no significant difference between the EA subjects and EC subjects (Figure S1B, PERMANOVA, *P* = 0.37) or between NEA and NEC subjects (Figure S1D, PERMANOVA, *P* = 0.73). These results suggested that within asthma and COPD, respectively, the fungal microbiota was dissimilar in subjects with different inflammatory phenotypes; however, the fungal microbiota of asthma and COPD subjects with the same inflammatory phenotype might be similar.

**Figure 3:**
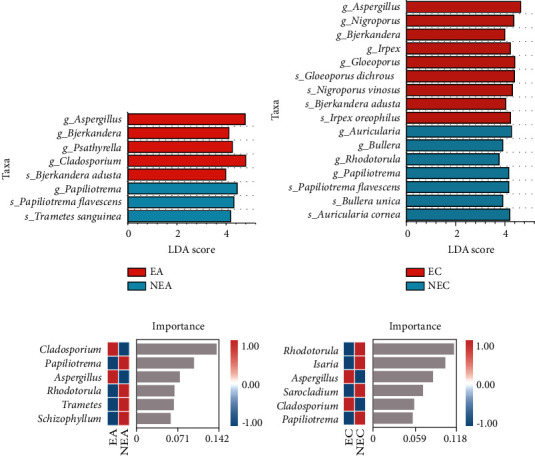
Differential taxa of fungi between eosinophilic and noneosinophilic inflammatory phenotypes. At genus and species levels, the LEfSe analysis between (a) EA vs. NEA and (b) EC vs. NEC. The LDA threshold was set at 3.0, and taxa with relative abundance less than 0.5% were filtered out. The fungal genera predicted by random forest analysis for classifying (c) asthma and (d) COPD subjects into eosinophilic and noneosinophilic inflammatory phenotypes. Random forest models were established against a 10-fold cross-validation.

The LEfSe analysis identified significant differences in 29 taxa between EA and NEA subjects (Figure S2A). At the genus and species levels (Figure 3(a)), *Aspergillus*, *Cladosporium*, *Psathyrella*, *and Bjerkandera adusta* exhibited higher abundances in EA subjects, whereas *Papiliotrema flavescens* and *Trametes sanguinea* exhibited significantly higher abundances in NEA subjects.

Additionally, 38 differential taxa were identified between EC and NEC subjects (Figure S2B). At the genus and species levels (Figure 3(b)), *Aspergillus*, *Gloeoporus dichrous*, *Irpex oreophilus*, *Nigroporus vinosus*, and *Bjerkandera adusta* were significantly enriched in EC subjects, whereas *Rhodotorula*, *Auricularia cornea*, *Bullera unica*, and *Papiliotrema flavescens* were greater in NEC subjects. Intriguingly, we found that not only *Aspergillus* and *Bjerkandera adusta* were enriched in EC subjects but also *Papiliotrema flavescens* were enriched in NEC subjects, which were same as asthmatic subjects.

Random forest models were applied to supplement the differential microbiota features of different inflammatory phenotypes in both asthma and COPD subjects. At the genus level, the overall accuracy was 0.87 (accuracy ratio = 1.50) for asthma subjects and 0.80 (accuracy ratio = 1.40) for COPD subjects (Figures 3(c) and 3(d)). The models indicated that *Aspergillus*, *Cladosporium*, *Papiliotrema*, and *Rhodotorula* are the common genera to distinguish eosinophilic and noneosinophilic inflammation, whether in subjects with asthma or COPD. The relative abundances of these genera varied significantly in Wilcoxon rank-sum test (*P* < 0.05), except for *Cladosporium*. *Cladosporium* had significantly greater relative abundances in EA subjects compared with NEA subjects (*P* = 0.002); however, this difference has not been observed in COPD subjects (*P* = 0.169).

Combining the results of LEfSe analysis and random forest models, *Aspergillus* and *Bjerkandera* were considered as fungal biomarkers of eosinophilic subjects in both asthma and COPD; meanwhile, *Papiliotrema* and *Rhodotorula* were regarded as biomarkers for noneosinophilic subjects.

### 5.3. Bacterial Microbiota

**Figure 4:**
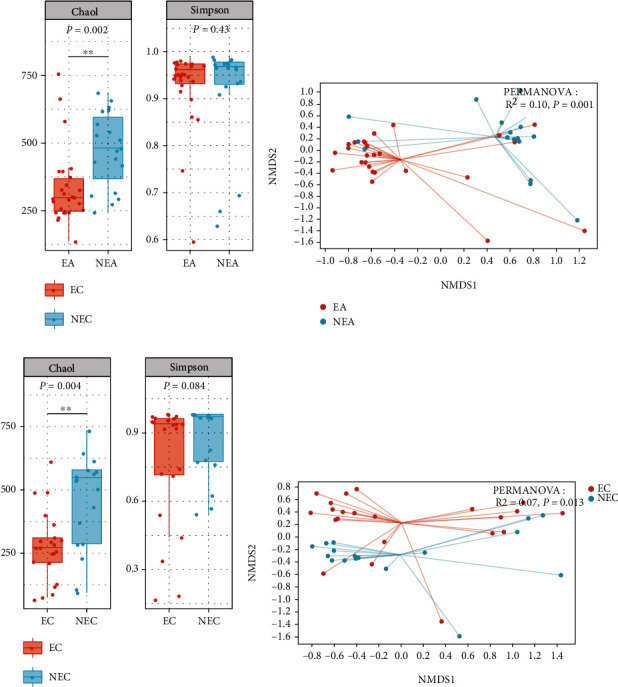
Alpha diversity and beta diversity of bacterial microbiota. Each dot represented a sample. Box plots of the Chao1 index and Simpson index, to compare alpha diversity (a) between EA and NEA and (c) between EC and NEC. The *y*-axis represented the value of the corresponding Chao1 or Simpson index. The statistical analysis was performed using Wilcoxon rank-sum test and Dunn' post hoc test. NMDS analysis based on the Bray-Curtis distance to compare beta diversity (b) between EA and NEA and (d) between EC and NEC. PERMANOVA was employed to reveal beta diversity variations.

In this study, NEA subjects showed higher *α*-diversity of bacteria compared with EA subjects (Figure 4(a), Chao1 index, *P* = 0.002), which coincided with the results of recent studies [8, 25]. Compared with EC subjects, the NEC subjects also had significantly increased Chao1 (Figure 4(c), *P* = 0.004) index. Moreover, EC subjects showed lower Shannon and Simpson indexes than EA subjects, however, not significantly, and there was no significant difference in *α*-diversity index between subjects of NEA and NEC (Figures S3A and S3C).

Significant difference was observed in *β*-diversity between EA and NEA subjects (Figure 4(b), PERMANOVA, *R*^2^ = 0.1, *P* = 0.001) and EC and NEC subjects (Figure 4(d), PERMANOVA, *R*^2^ = 0.07, *P* = 0.013). That result indicated that eosinophilic and noneosinophilic subjects had different bacterial microbiota within asthma and COPD, respectively. Furthermore, in the comparison between asthma and COPD subjects with eosinophilic inflammation, significant difference was found between EC subjects and EA subjects (Figure S3B, PERMANOVA, *R*^2^ = 0.05, *P* = 0.012). By contrast, we did not find a distinction in *β*-diversity between asthma and COPD subjects with noneosinophilic inflammation (Figure S3D, PERMANOVA, *P* = 0.26), but the effect of large intersample variability on this outcome was also a nonnegligible factor that needs to be further evaluated in subsequent studies.

**Figure 5:**
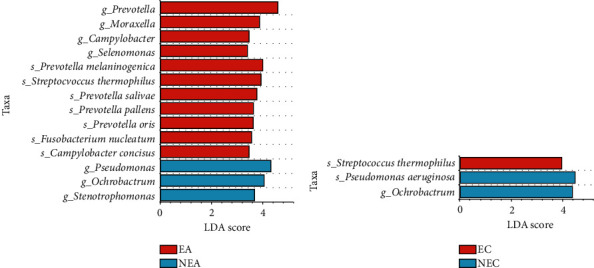
Differential taxa of bacteria between eosinophilic and noneosinophilic inflammatory phenotypes. At genus and species levels, the LEfSe analysis between (a) EA vs. NEA and (b) EC vs. NEC. The LDA threshold was set at 3.0, and taxa with relative abundance less than 0.5% were filtered out.

Using LEfSe analysis, we identified 34 differential taxa between EA and NEA subjects (Figure S4A). At the genus and species levels (Figure 5(a)), EA subjects were enriched in *Moraxella*, *Selenomonas*, *Fusobacterium nucleatum, Prevotella salivae, Prevotella pallens*, *Prevotella oris*, *Prevotella melaninogenica*, *Streptococcus thermophilus*, and *Campylobacter concisus*, whereas NEA subjects were enriched in *Pseudomonas*, *Ochrobactrum*, and *Stenotrophomonas*. In particular, *Ochrobactrum* and *Streptococcus thermophilus* had also been differential taxa in the comparison between EC and NEC subjects (Figure 5(b)).

### 5.4. Fungal-Bacterial Correlation Network Analysis

**Figure 6:**
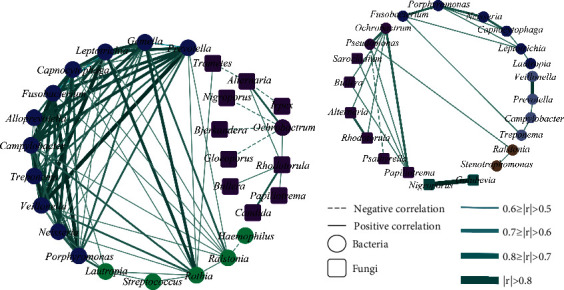
Fungal-bacterial correlation network analysis at genus level in (a) COPD and (b) asthma. Each microbial taxon represented by a node, the round nodes represent the genus of bacteria, the square nodes represent the genus of fungi. The network represents significant Spearman's correlation coefficients between the top 20 fungal and bacterial genera (FDR-corrected *P* < 0.01, ∣*r* | >0.5). The solid line indicated positive correlation, the dashed line indicated negative correlation, and the strength of correlation between genera was indicated by the dark and light colors and the thickness of the lines. The color of the nodes represented the different modules.

To investigate the similarities and differences in bacterial-fungal interactions between COPD (Figure 6(a)) and asthma (Figure 6(b)), we performed a correlation network analysis. Overall, COPD subjects demonstrated greater connection density compared to asthma subjects. Interestingly, we observed some similar correlations in asthma subjects and used the same colors to represent these modules. The blue module of COPD and asthma included *Fusobacterium*, *Porphyromonas*, *Neisseria*, *Capnocytophaga*, and *Leptotrichia*. Furthermore, the *Ochrobactrum*, *Alternaria*, *Rhodotorula*, *Papiliotrema* and *Bullera* also appear in the purple module of the asthma subjects. However, as only a small number of correlations were observed, we could not construct a valid network to compare differences between samples with different inflammatory phenotypes.

## 6. Discussion

In this study, we collected induced sputum samples from 45 asthma subjects and 39 COPD subjects to explore the microbiota differences between eosinophilic and noneosinophilic phenotypes. We reported that the fungal microbiota of airway eosinophilic and noneosinophilic inflammatory phenotypes in COPD subjects was different, and the NEC subjects showed higher fungal *α*-diversity and *β*-diversity than EC subjects. Similarly, the EA and NEA subjects had considerable differences in fungal microbiota; this result was similar to the previous study from asthma subjects [10, 26]. Interestingly, our research revealed that the fungal taxa associated with eosinophilic or noneosinophilic inflammation were consistent in both asthma and COPD.

Several studies demonstrated that fungal colonization can contribute to airway inflammatory diseases as an important allergen [27–30]. *Aspergillus*, *Cladosporium*, and *Rhodotorula* were previously shown to be present in healthy individuals, and their relative abundance was significantly altered in asthma patients and associated with Th2 inflammation [10, 25]. Among the asthma subjects in this study, EA subjects had higher relative abundances in *Aspergillus*, *Cladosporium*, *Psathyrella*, and *Bjerkandera adusta* species. *Cladosporium* is known to cause lung allergic inflammation, airway hyperreactivity, and remodeling in mice [31], and *Aspergillus* also play a momentous role in airway inflammatory and allergic diseases as a type 2 inflammation adjuvant [32]. In addition, it was being provided that *Bjerkandera* can lead to eosinophilic infiltration in the airways along with Th2 cytokine and eosinophil-related chemokine production [33, 34]. However, studies on the fungal microbiota of COPD patients are still scarce. We reported that *Aspergillus*, *Gloeoporus dichrous*, *Irpex oreophilus*, *Nigroporus vinosus*, and *Bjerkandera adusta* were associated with higher sputum eosinophils in COPD subjects. Our study indicated that *Aspergillus* and *Bjerkandera* were significantly enriched in subjects with eosinophilic inflammation, and *Rhodotorula* and *Papiliotrema* had higher relative abundances in subjects with noneosinophilic inflammation, both in asthma and COPD.

Previous studies had demonstrated that asthma subjects with higher eosinophilic inflammation showed lower bacterial diversity than the subjects with lower eosinophilic inflammation [8, 35, 36]. In our study, obvious differences also emerged in the bacterial microbiota of COPD subjects. In healthy individuals, a core bacterial microbiota dominated by *Streptococcus*, *Prevotella*, *Veillonella*, *Pseudomonas*, *Haemophilus*, and *Fusobacterium* has been reported [37]. In asthma subjects of this study, the EA subjects were enriched in 4 species from *Prevotella* (*Prevotella oris*, *Prevotella salivae*, *Prevotella melaninogenica*, and *Prevotella pallens*) and *Fusobacterium nucleatum*, *Campylobacter concisus*, *Streptococcus thermophilus*, *Moraxella*, and *Selenomonas*, while the NEA subjects had higher abundance in *Pseudomonas* and *Ochrobactrum*. Moreover, we observed some comparable species in COPD subjects, such as *Streptococcus thermophilus* also enriched in EC subjects and *Ochrobactrum* and *Pseudomonas aeruginosa* enriched in NEC subjects. *Prevotella*, *Fusobacterium*, and *Selenomonas* were demonstrated by a previous study to be enriched in atopic asthma subjects [35]; our results showed these species were also associated with sputum eosinophils. Wang et al. [5] demonstrated that *Prevotella aurantiaca* and *Fusobacterium nucleatum* exhibited positive associations with Th2-related mediators in COPD patients, and we further observed the enrichment of multiple species belonging to Prevotella and Fusobacterium in asthma patients with eosinophilic phenotype. In contrast, *Pseudomonas* could increase the regulatory T cell response and inhibited concomitant Th2 response [38], so the enrichment of *Pseudomonas aeruginosa* was observed in the noneosinophilic group. Another genus, *Streptococcus*, was known to exhibit greater abundance in asthma subjects with an eosinophilic inflammatory phenotype [36], in COPD subjects *Streptococcus* was positively correlated with blood eosinophil counts [9]; our study similarly supported the association of *Streptococcu*s with airway eosinophilic inflammation.

Previous studies have suggested an important role for microbial interactions in asthma, which associated with airway inflammation and disease outcome [10, 39]. As representatives of airway inflammatory diseases, asthma and COPD have some overlaps in the airway bacterial microbiota, and we found that they share a degree of homogeneity in microbial correlations. Liu et al. [25] demonstrated significant correlations in patients with asthma between *Prevotella*, *Veillonella*, *Leptotrichia*, and *Rothia*; our study further illustrated similar correlations in COPD patients as well. Furthermore, the significant positive correlations were observed between *Ochrobactrum*, *Alternaria*, and *Rhodotorula* in both asthma and COPD. In the debate between the “British” and “Dutch” hypothesis [40], we provided a perspective from the interaction of airway inflammatory phenotypes and microbiota structure; asthma and COPD have both common and separate features. Understanding the interaction is the first step towards clinical application, and further clarification of the mechanisms behind the interaction results will help us to better improve clinical practice.

There are some potential confounders and limitations that need to be considered in our study. Firstly, limited by the relatively small sample size of the current study, the results require the support of a larger sample study. Secondly, since the fungal database needs to be improved compared to the bacterial database and to obtain more reliable annotation information, we set the BROCC algorithm threshold to 100%, so the taxonomic classification efficiency of fungi was lower than that of bacteria. Additionally, the smoking rates and ICS doses differed between asthma and COPD patients in this study, the effect of smoking and ICS on airway microbiota had been demonstrated by previous studies [35, 41, 42], and that influence to this result needs to be explored in the follow-up study.

## 7. Conclusions

This study emphasized that fungal and bacterial microbiota were associated with a specific airway inflammatory context and revealed the overlaps of differential microbiota features in particular phenotypes between subjects with asthma and COPD. Our study highlighted the heterogeneity within asthma and COPD, respectively, and the homogeneity between them, guiding further research into disease pathogenesis and the development of potential targeted therapies.

## Figures and Tables

**Table 1:** Enrolled subjects' characteristics.

Parameters	Asthma		COPD		*P* value			
	EA (*n* = 26)	NEA (*n* = 19)	EC (*n* = 22)	NEC (*n* = 17)	EA vs. NEA	EC vs. NEC	EA vs. EC	NEA vs. NEC
Male (%)	9 (34.62)	8 (42.11)	21 (95.45)	14 (82.35)	0.75^‡^	0.3^‡^	**0.005** ^‡^	**<0.001** ^‡^
Age (years)	54.04 ± 11.41	57.68 ± 12.11	68.55 ± 7.53	71 ± 5.97	0.308^†^	0.21^※^	**<0.001** ^†^	**<0.001** ^※^
BMI (kg/m^2^)	23.02 ± 3.56	23.59 ± 3.22	23.37 ± 2.50	24.12 ± 3.06	0.455^※^	0.406^†^	0.463^※^	0.617^†^
ICS dose (*μ*g/d)	417.31 ± 282.47	657.89 ± 346.92	890.9 ± 236.86	935.29 ± 183.51	**0.025** ^※^	0.705^※^	**<0.001** ^※^	**0.025** ^※^
Smoking (%)	7 (26.92)	6 (31.58)	15 (68.18)	11 (64.71)	0.751^‡^	1^‡^	**0.008** ^‡^	0.093^‡^
FEV_1_ (%predicted)	71.68 ± 14.97	75.22 ± 24.66	43.38 ± 10.23	43.07 ± 13.35	0.455^※^	0.216^※^	**<0.001** ^†^	**<0.001** ^※^
FEV_1_/FVC (%)	61.17 ± 11.93	60.75 ± 19.03	40.55 ± 7.64	44.02 ± 9.58	0.696^※^	0.305^†^	**<0.001** ^※^	**<0.001** ^※^
Sputum eosinophils (%)	20.9 ± 24.62	1.14 ± 0.83	15.26 ± 22.93	1.05 ± 0.82	**<0.001** ^※^	**<0.001** ^※^	0.444^※^	0.739^†^
Sputum neutrophils (%)	57 ± 56.58	81.89 ± 7.64	65.91 ± 24.69	88.23 ± 6.75	**0.001** ^※^	**<0.001** ^※^	0.264^※^	**0.013** ^†^
ACT score	21.42 ± 3.28	21.74 ± 5.26	—	—	0.954^※^	—	—	—
CAT score	—	—	19.05 ± 6.3	18.64 ± 6.54	—	0.848^†^	—	—

Data were presented as *n* (%) and mean ± SD unless otherwise stated. The ICS doses were converted to equivalents of fluticasone. ^†^Tested by *t*-test. ^‡^Tested by Fisher's exact test. ^※^Tested by Wilcoxon rank-sum test.

## Data Availability

The sequencing datasets of this study can be found in the NCBI under accession number PRJNA751382.

## Supplementary Materials

Supplementary Materials Table S1: the overview of microbiota profile. Table S2: the top 5 genera of bacterial relative abundance. Table S3: the top 5 genera of fungal relative abundance. Figure S1: alpha and beta diversity of fungal microbiota. Figure S2: differential taxa of fungi between eosinophilic and noneosinophilic inflammation in asthma (A) and COPD (B). FigureS3: alpha and beta diversity of bacterial microbiota. Figure S4: differential taxa of bacteria between eosinophilic and noneosinophilic inflammation in asthma (A) and COPD (B). Figure S5: NMDS plots and Venn plots containing samples from each group.
