# Supplementary material for: Different Airway Inflammatory Phenotypes Correlate with Specific Fungal and Bacterial Microbiota in Asthma and Chronic Obstructive Pulmonary Disease
Source: J Immunol Res. 2022 Mar 11;2022:2177884. doi: 10.1155/2022/2177884 (PMC8933093; doi:10.1155/2022/2177884)
Supplement: Supplementary Materials — Table S1: the overview of microbiota profile. Table S2: the top 5 genera of bacterial relative abundance. Table S3: the top 5 genera of fungal relative abundance. Figure S1: alpha and beta diversity of fungal microbiota. Figure S2: differential taxa of fungi between eosinophilic and noneosinophilic inflammation in asthma (A) and COPD (B). FigureS3: alpha and beta diversity of bacterial microbiota. Figure S4: differential taxa of bacteria between eosinophilic and noneosinophilic inflammation in asthma (A) and COPD (B). Figure S5: NMDS plots and Venn plots containing samples from each group. [file 2177884.f1.docx]

**Supplementary**

**Table S1. The overview of microbiota profile.**

| **Taxa** | **Bacteria** | **Fungi** |
| --- | --- | --- |
| Phyla | 16 | 2 |
| Genera | 186 | 71 |
| Species | 181 | 61 |
| OTUs | 3346 | 719 |
| Resampling depth | 3267 | 1776 |
| Total number of  non-singleton reads  (high-quality reads) | 589521 | 386269 |

**Table S2. The top 5 genera of bacterial relative abundance.**

| Asthma | | COPD | |
| --- | --- | --- | --- |
| EA (n=26) | NEA (n=19) | EC (n=22) | NEC (n=17) |
| *Prevotella* (13.79 %) | *Streptococcus* (14.01 %) | *Haemophilus* (27.22 %) | *Haemophilus* (19.21 %) |
| *Neisseria* (11.99 %) | *Haemophilus* (12.41 %) | *Streptococcus* (15.08 %) | *Streptococcus* (10.04 %) |
| *Haemophilus* (11.55 %) | *Neisseria* (9.33 %) | *Prevotella* (7.15 %) | *Pseudomonas* (8.82 %) |
| *Streptococcus* (9.75 %) | *Pseudomonas* (6.48%) | *Neisseria* (6.4 %) | *Moraxella* (8.5 %) |
| *Porphyromonas* (4.22 %) | *Prevotella* (6.29 %) | *Pseudomonas* (5.04 %) | *Neisseria* (7.80 %) |

**Table S3. The top 5 genera of fungal relative abundance.**

| Asthma | | COPD | |
| --- | --- | --- | --- |
| EA (n=26) | NEA (n=19) | EC (n=22) | NEC (n=17) |
| *Aspergillus* (22.32 %) | *Aspergillus* (8.89 %) | *Aspergillus* (11.89 %) | *Candida* (8.64 %) |
| *Candida* (13.20 %) | *Trametes* (7.64 %) | *Cladosporium* (9.59 %) | *Cladosporium* (4.48 %) |
| *Cladosporium* (12.78 %) | *Schizophyllum* (5.93 %) | *Malassezia* (6.61 %) | *Malassezia* (4.07 %) |
| *Malassezia* (4.65 %) | *Cladosporium* (1.8%) | *Candida* (5.07 %) | *Aspergillus* (4.01 %) |
| *Psathyrella* (2.62 %) | *Papiliotrema* (1.65 %) | *Trametes* (4.32 %) | *Papiliotrema* (2.79 %) |


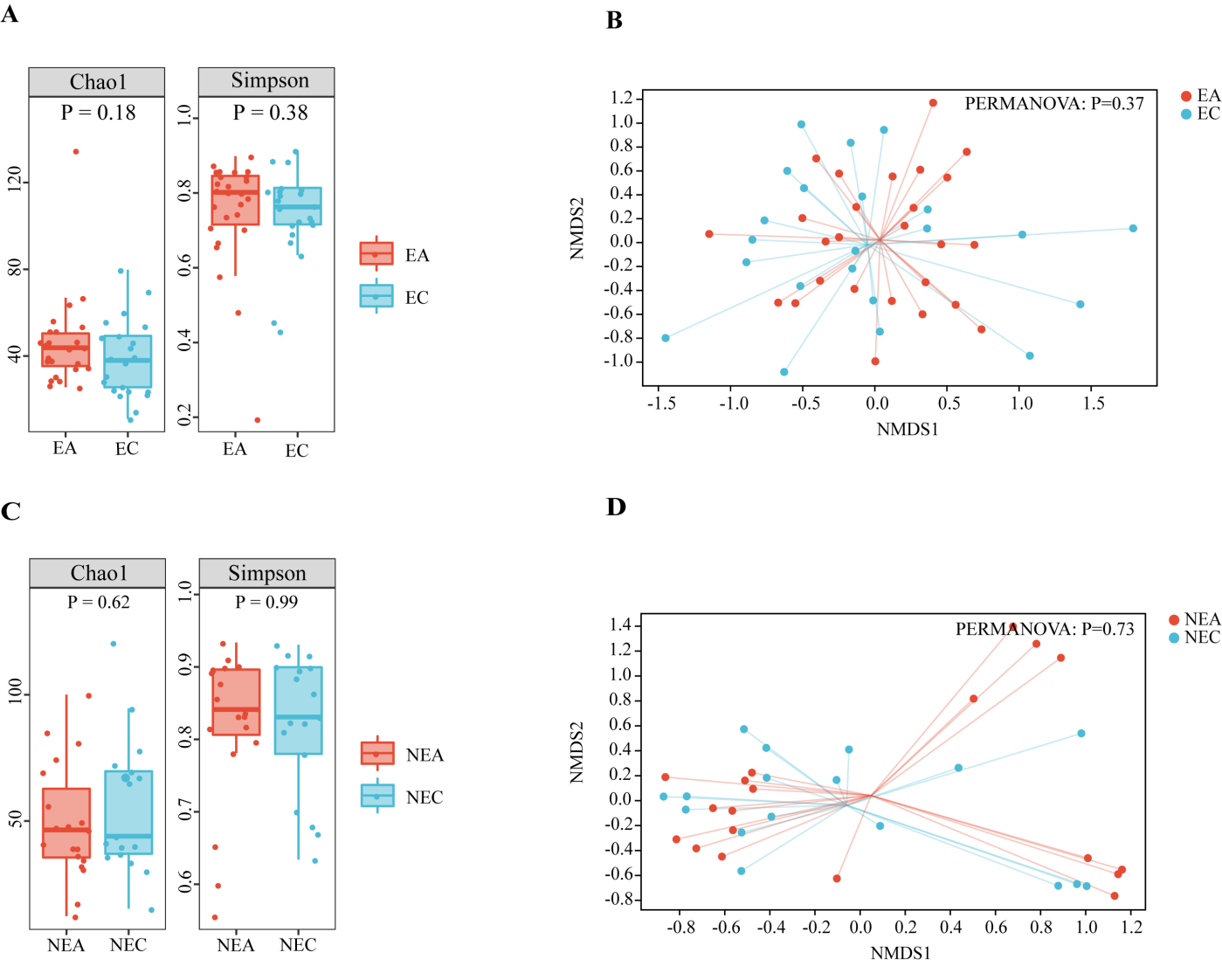


**Figure S1.** **Alpha and beta diversity of fungal microbiota.** Each dot represented a sample. **A** and **C**, box plots of the Chao1 index and Simpson index, to compare alpha diversity between EA and EC (A), between NEA and NEC (C). The Y-axis represented the value of the corresponding Chao1 or Simpson index. The statistical analysis was performed using Wilcoxon rank-sum test and Dunn' post hoc test. **B** and **D**, NMDS analysis based on the Bray-Curtis distance to compare beta diversity between EA and EC (B) and between NEA and NEC (D). PERMANOVA was employed to reveal beta diversity variations.


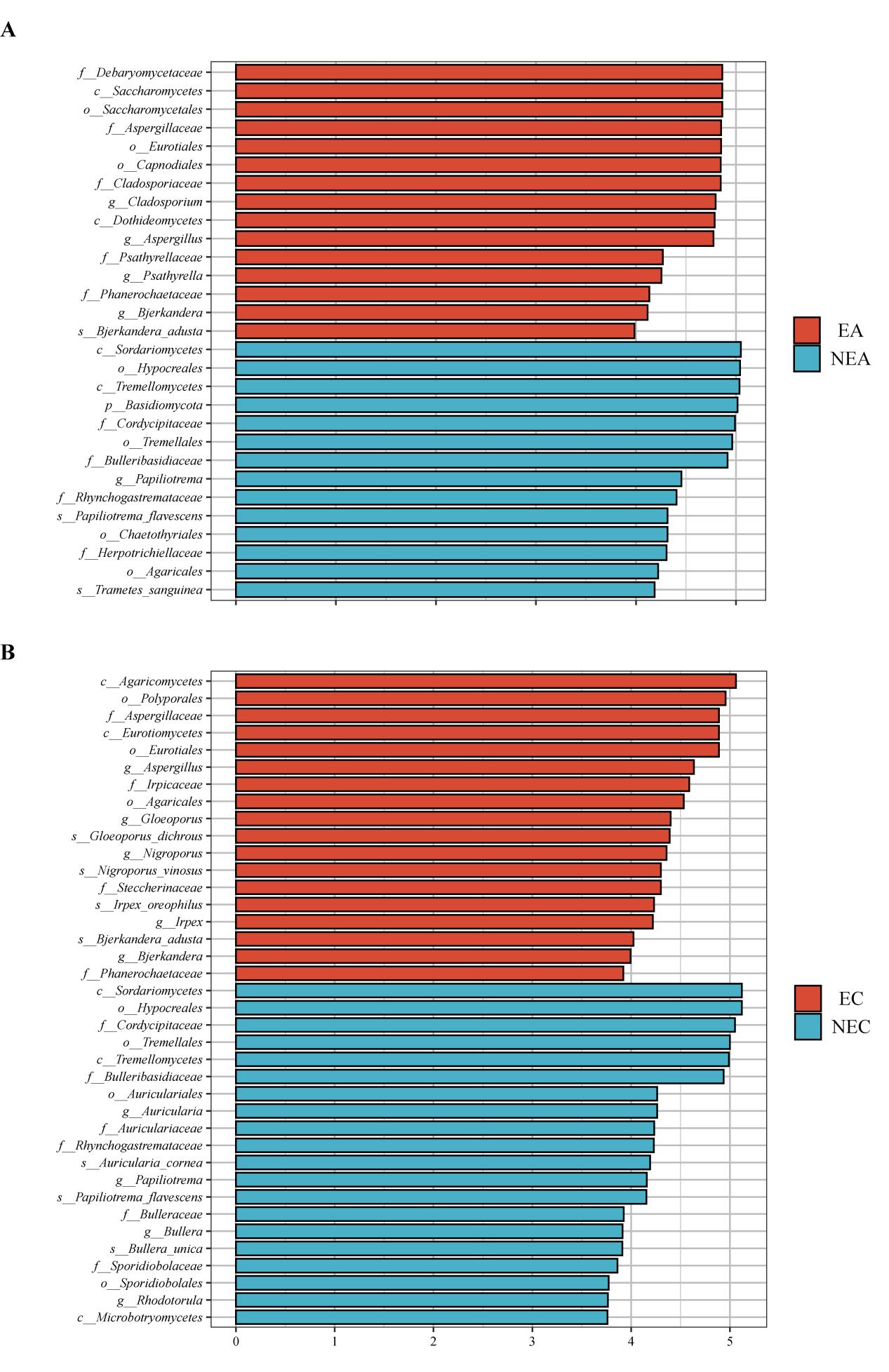


**Figure S2. Differential taxa of fungi between eosinophilic and non-eosinophilic inflammation in asthma (A) and COPD (B).** The LDA threshold was set at 3.0, and taxa with relative abundance less than 0.5% were filtered.


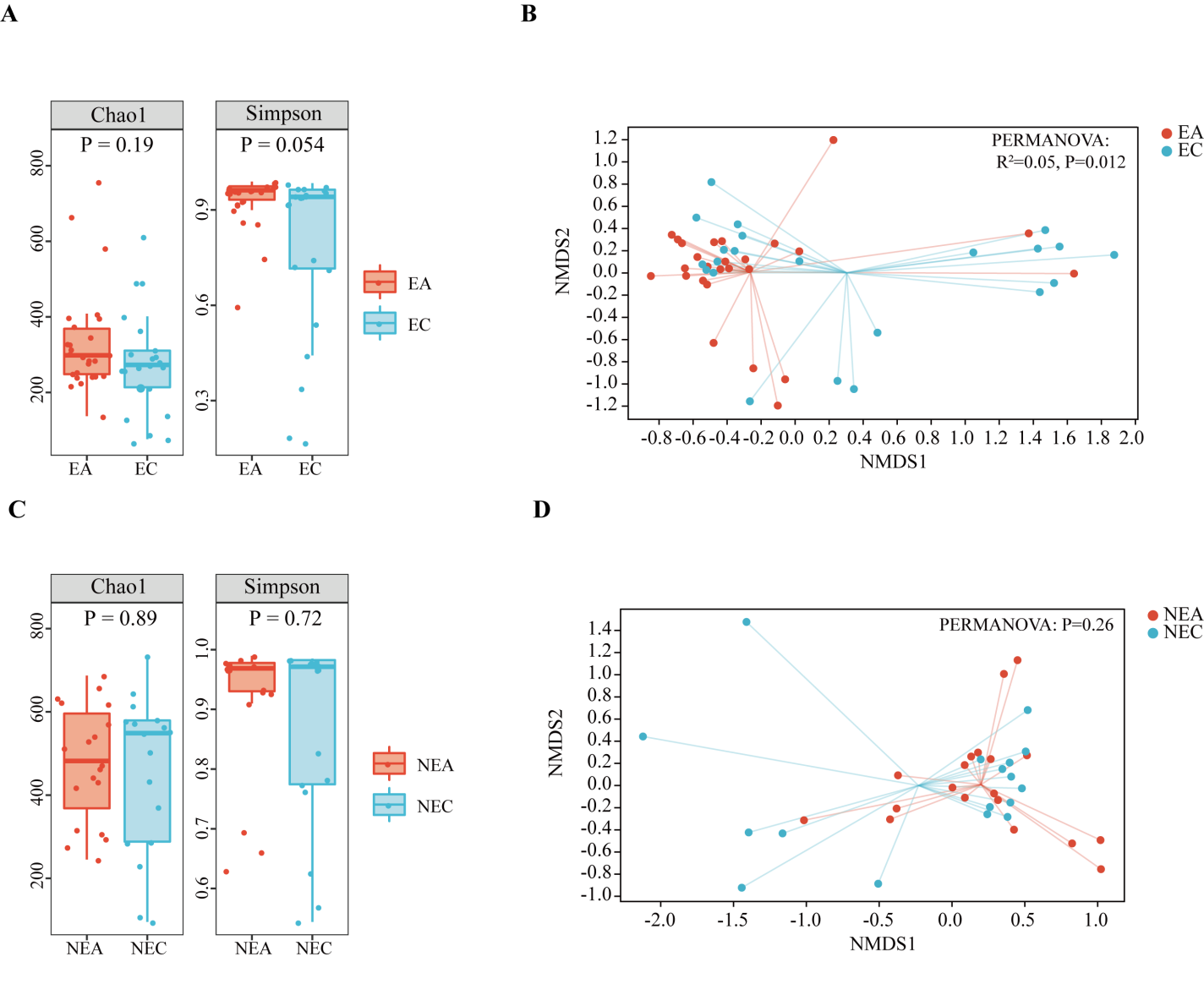


**Figure** **S3.** **Alpha and beta diversity of bacterial microbiota.** Each dot represented a sample. **A** and **C**, box plots of the Chao1 index and Simpson index, to compare alpha diversity between EA and EC (A), between NEA and NEC (C). The Y-axis represented the value of the corresponding Chao1 or Simpson index. The statistical analysis was performed using Wilcoxon rank-sum test and Dunn' post hoc test. **B** and **D**, NMDS analysis based on the Bray-Curtis distance to compare beta diversity between EA and EC (B) and between NEA and NEC (D). PERMANOVA was employed to reveal beta diversity variations.


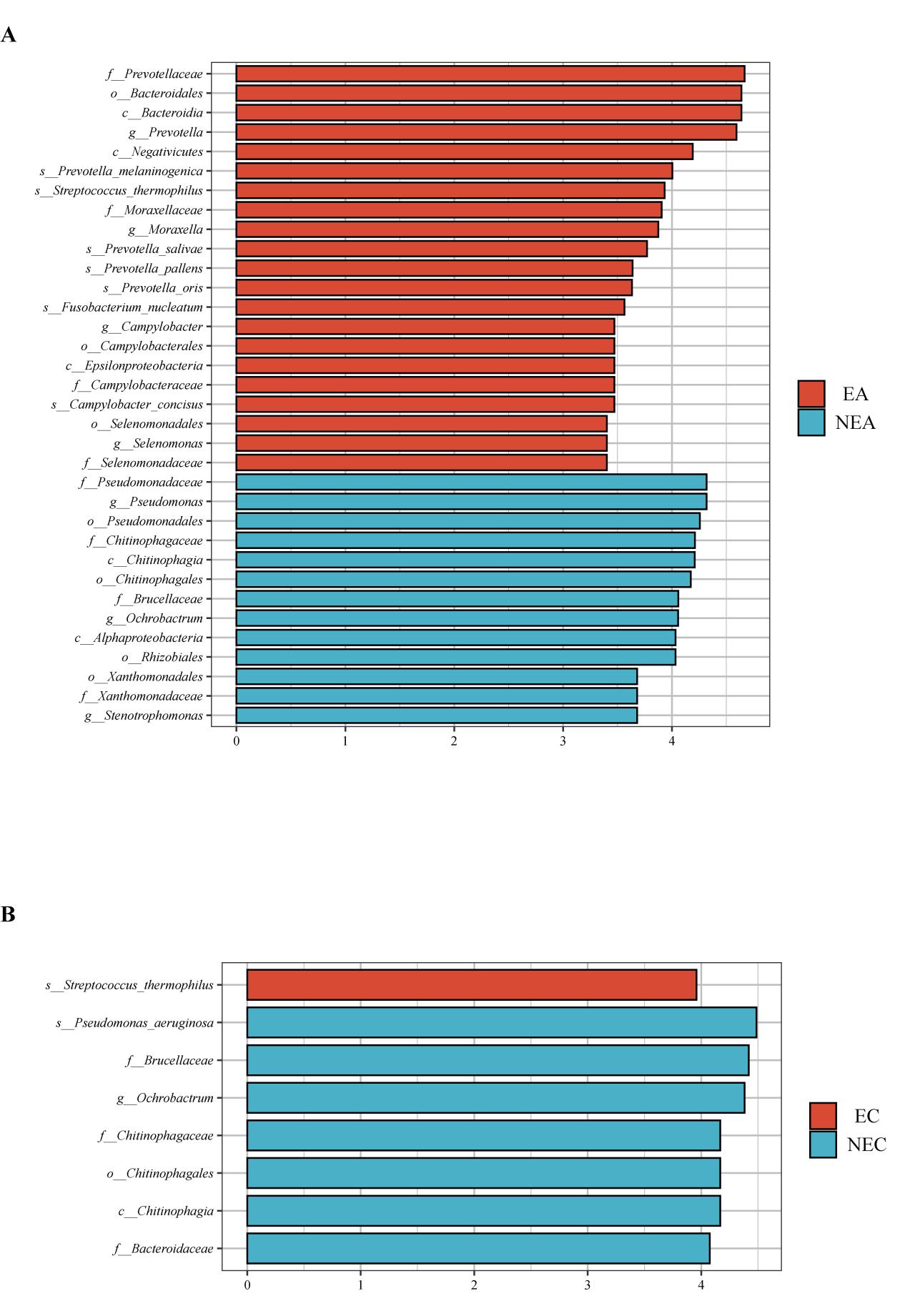


**Figure S4. Differential taxa of bacteria between eosinophilic and non-eosinophilic inflammation in asthma (A) and COPD (B).** The LDA threshold was set at 3.0, and taxa with relative abundance less than 0.5% were filtered out.


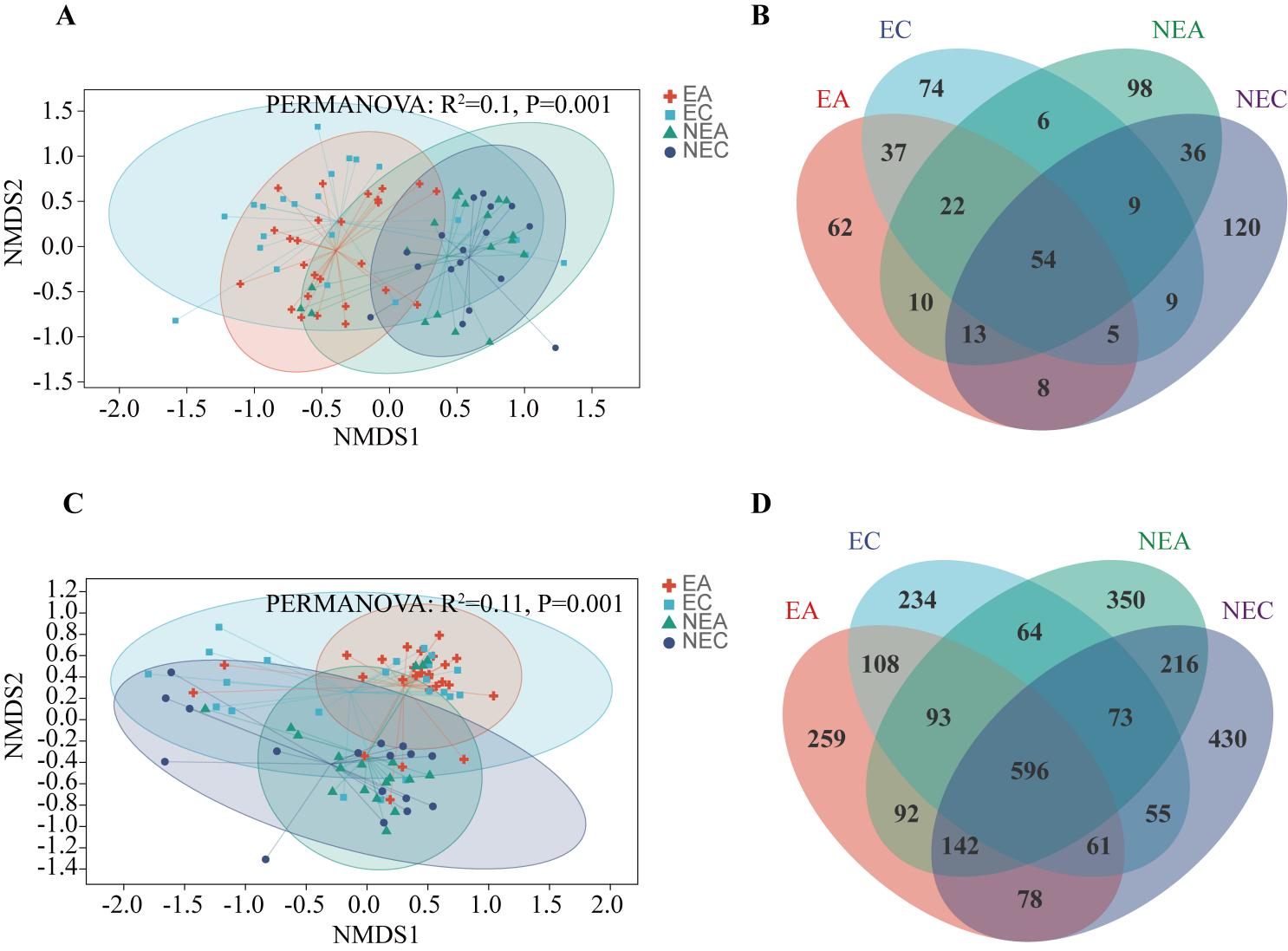


**Figure S5. NMDS plots and Venn plots containing samples from each group. A** and **C**, NMDS analysis of fungal (**A**) and bacterial (**C**) microbiota based on the Bray-Curtis distance. Each dot represented a sample. PERMANOVA was employed to reveal beta diversity variations of all four groups. **C** and **D**, Venn plots for revealing the common fungal (**B**) and bacterial OTUs (**D**) between groups. Each color block represented a group, the overlapping area indicated the common OTUs of the corresponding group, and the number of each block indicated the number of OTUs contained in that group.
